# The stability of the Autism Diagnostic Observation Schedule‐2 in children aged 14–36 months with elevated likelihood for autism

**DOI:** 10.1111/jcpp.70078

**Published:** 2025-11-16

**Authors:** Sarah Schaubroeck, Ellen Demurie, Jannath Begum‐Ali, Sven Bölte, Sofie Boterberg, Jan Buitelaar, Tony Charman, Terje Falck‐Ytter, Sabine Hunnius, Mark H. Johnson, Emily Jones, Iris Oosterling, Greg Pasco, Mirjam Pijl, Carlijn Van den Boomen, Petra Warreyn, Herbert Roeyers

**Affiliations:** ^1^ Research in Developmental Diversity Lab (RIDDL), UGent, Department of Experimental Clinical and Health Psychology Ghent University Ghent Belgium; ^2^ Centre for Brain and Cognitive Development, School of Psychological Sciences, Faculty of Science, Birkbeck University of London London UK; ^3^ Center of Neurodevelopmental Disorders (KIND), Centre for Psychiatry Research, Department of Women's and Children's Health, Karolinska Institutet & Stockholm Health Care Services, Region Stockholm Stockholm Sweden; ^4^ Curtin Autism Research Group, School of Allied Health Curtin University Perth WA Australia; ^5^ Child and Adolescent Psychiatry, Stockholm Health Care Services Stockholm Sweden; ^6^ Department of Medical Neuroscience, Donders Institute for Brain, Cognition and Behavior Radboud University Medical Centre Nijmegen The Netherlands; ^7^ Karakter Child and Adolescent Psychiatry University Centre Nijmegen The Netherlands; ^8^ Department of Psychology Institute of Psychiatry, Psychology & Neuroscience, King's College London London UK; ^9^ Development and Neurodiversity Lab, Department of Psychology Uppsala University Uppsala Sweden; ^10^ Donders Centre for Cognition, Donders Institute for Brain, Cognition & Behaviour Radboud University Nijmegen The Netherlands; ^11^ Department of Psychology University of Cambridge Cambridge UK; ^12^ Centre for Developmental Neurobiology, Institute of Psychiatry, Psychology & Neuroscience Kings College London London UK; ^13^ Department of Child and Adolescent Psychiatry, Institute of Psychiatry, Psychology & Neuroscience Kings College London London UK; ^14^ Experimental Psychology Helmholtz Institute Utrecht University Utrecht The Netherlands

**Keywords:** Autism, stability, ADOS, diagnosis, EL‐siblings

## Abstract

**Background:**

This study investigated the stability of Autism Diagnostic Observation Schedule, Second Edition (ADOS‐2) classifications in a cohort of 304 siblings at elevated likelihood for autism (EL‐siblings).

**Methods:**

ADOS‐2 assessments were conducted at 14, 24 and 36 months, with Clinical Best Estimate (CBE) autism diagnoses determined at 36 months.

**Results:**

Our findings indicate that while some children have stable ADOS‐2 classifications from early on, a significant proportion of the children show inconsistent classifications over time. The overall stability of ADOS‐2 autism spectrum classifications increased from 14 to 36 months and agreement with CBE autism clinical diagnosis was moderate and increased with age.

**Conclusions:**

Caution is warranted when interpreting individual ADOS‐2 results, as they should always complement, and can never replace, a comprehensive clinical evaluation. These findings highlight the importance of continued follow‐up beyond 14 months in young EL‐children, a group for whom early assessment may be both feasible and beneficial and emphasises the need to integrate multiple assessment measures and multiple informants for accurate early autism identification.

## Introduction

Autism is a neurodevelopmental condition with a prevalence of about 1%–2% (Maenner et al., 2023; Zeidan et al., 2022). It is characterised by challenges in social communication and interaction, alongside restricted and repetitive behaviours, interests or activities and sensory challenges (American Psychiatric Association [APA], 2022). Children with an older autistic sibling have a significantly elevated likelihood (EL) to be autistic themselves. Sibling studies show that 10%–20% of children with an autistic sibling are diagnosed with autism by age 3 (Ozonoff et al., 2024). This elevated likelihood is further supported by large population studies reporting rates around 10% (Hansen et al., 2019; Jokiranta‐Olkoniemi et al., 2016; Sandin, Lichtenstein, Kuja‐Halkola, Hultman, & Reichenberg, 2017). Consequently, prospective research in infant EL siblings provides the opportunity to study the early development of autistic children (van 't Hof et al., 2021).

The identification of autism at an early age is challenging due to phenotypic heterogeneity, including differences in the timing of feature onset and behavioural manifestations among autistic children (Kim, Macari, Koller, & Chawarska, 2016). Moreover, co‐occurring difficulties or conditions, such as intellectual disability, atypical language development and attention‐deficit/hyperactivity disorder (ADHD) complicate the diagnostic process in EL siblings (Charman et al., 2017, 2023; Marrus et al., 2018; Messinger et al., 2013). However, early identification of autism characteristics and subsequent diagnostic classification is crucial for recognising potential challenges and facilitating early support or targeted intervention (Fuller & Kaiser, 2020; Ryberg, 2015). In turn, this might prevent the development of secondary difficulties and improve long term outcomes and quality of life (Turner, Stone, Pozdol, & Coonrod, 2006). Given these potential advantages, timely recognition of autism characteristics in EL‐siblings is essential. In clinical practice, administering the Autism Diagnostic Observation Schedule‐2 (ADOS‐2; Lord et al., 2012), often combined with the Autism Diagnostic Interview‐Revised (ADI‐R; Rutter, Le Couteur, & Lord, 2003), is standard procedure in assessing autism characteristics. While an autism diagnosis must always be grounded in comprehensive clinical judgement rather than relying solely on the ADOS‐2, the instrument provides standardised information to support expert clinician evaluations (Bishop & Lord, 2023; Fombonne, 2023). Considering this, it is important to examine whether ADOS‐2 classifications remain stable across development, not because repeated administration should function as a standalone diagnostic pathway, but because such stability can provide insight into how the tool contributes to broader developmental monitoring.

Research by Ozonoff et al. (2015) and Zwaigenbaum et al. (2016) has examined diagnostic stability in EL‐siblings and indicated that a reliable and stable clinical diagnosis of autism can be established between 18 and 24 months, although this can vary depending on individual factors, such as developmental level, presence of autistic characteristics or sex (Lai, Lombardo, Auyeung, Chakrabarti, & Baron‐Cohen, 2015; Zwaigenbaum et al., 2015). Ozonoff et al. (2015) found that the stability of an autism diagnosis was 93% at 18 months and 82% at 24 months. However, many children with autism outcomes at 36 months were not diagnosed until later, with 63% not identified at 18 months. Similarly, Zwaigenbaum et al. (2015) reported that 82.6% of diagnoses made at 18 months were confirmed at 3 years, but a significant proportion (12.1%) were diagnosed at 3 years who had not been identified earlier. These findings highlight the importance of repeated assessments and ongoing monitoring, as many children may not meet diagnostic criteria at younger ages.

Research specifically examining the stability of ADOS‐2 classifications is limited. Although its psychometric properties are well established (De Bildt et al., 2009; Gotham et al., 2008; Gray, Bruce, Ae, & Sweeney, 2007; Kamp‐Becker et al., 2011; Le Couteur, Haden, Hammal, & McConachie, 2008; Oosterling et al., 2010), the longitudinal stability of ADOS‐2 classifications in EL‐siblings across early childhood has not been systematically studied. It is important to recognise that diagnostic decisions are based on comprehensive assessments that integrate multiple data sources, with the ADOS‐2 representing only one component (Bishop & Lord, 2023). Clarifying the stability of ADOS‐2 classifications over time contributes to the broader understanding of diagnostic stability and informs the clinical interpretation of ADOS‐2 classifications relative to other assessment modalities and tools. It is also important to note that this study was conducted with volunteer families with an older autistic child participating in a research study, not children who were clinically referred for developmental concerns. The assessment context therefore differs from clinical samples, where parents actively seek clarity on a diagnosis, which may influence diagnostic decisions and the agreement between the ADOS‐2 and Clinical Best Estimate (CBE) diagnosis. Research diagnoses were not communicated to families, but were used solely for scientific purposes, which further distinguishes the research context from clinical practice. With ADOS‐2 classifications, we refer specifically to the classifications of autism spectrum according to ADOS‐2 threshold.

The first research question addresses the stability of ADOS‐2 classifications over time (defined as being either consistently above or consistently below the ADOS‐2 autism spectrum cutoff at both time points). Although no prior research is available, we expected increasing stability in ADOS‐2 classifications with age. Second, we investigated whether sex and developmental levels differed between the ADOS‐2 classifications at each time point and between stable and unstable ADOS‐2 classification patterns over time. Based on previous literature, we expected boys and children with lower developmental levels to show more ADOS‐2 positive classifications (Loomes, Hull, & Mandy, 2017; Messinger et al., 2015). We also examined the agreement between ADOS‐2 classifications at 14, 24 and 36 m and CBE diagnosis at 36 m and analysed the ADOS‐2 classification patterns of children with an autism outcome. We expected that a subset of children would display stable ADOS‐2 classifications up to the age of 3. These children may already show a clear picture early on, with more developmental difficulties, evident by cognitive delay or more pronounced autism characteristics compared to children with unstable ADOS‐2 classifications (Chawarska et al., 2014; Zwaigenbaum et al., 2016).

## Method

### Participants

The sample included 304 EL‐siblings drawn from a prospective, longitudinal multi‐site study conducted by the EuroSibs Autism Research Network (Jones et al., 2019). Assessments were conducted at 5, 10, 14, 24 and 36 months (m, for months). Ethical approval was given by local or national ethics committees, following the Declaration of Helsinki and APA guidelines. Parents gave written consent. EL‐children had at least one older sibling with a community diagnosis of autism. Families were recruited through well‐baby clinics, child‐care and diagnostic centers, autism services and parent events. Exclusion criteria included epilepsy, preterm birth (i.e., <36 weeks gestational age) and genetic syndromes related to autism. EL‐data from four sites were used: the Early Autism Sweden (EASE) project (*n* = 221, Sweden), Babystudy project (*n* = 60, Belgium), Siblings of Children with Autism (ZEBRA) project (*n* = 52, the Netherlands) and Studying Autism and ADHD in the Early Years (STAARS) project (*n* = 101, UK). Participants were excluded if ADOS‐2 data were missing at two or more timepoints (*n* = 128). Missing data resulted from missed appointments, dropouts and unadministered instruments (e.g., ADOS‐2 at 14 m was excluded in the UK protocol and largely unavailable for Sweden). Missing data were not imputed, as this was deemed inappropriate for the study's aims. Analyses were based on available data; for an overview, see Table S1. Excluded participants did not differ significantly from the included sample in terms of sex or developmental level at 14 and 24 m but at 36 m excluded children had a lower developmental level than included children; however, only 24 excluded children had Mullen Scales of Early Learning (MSEL) data available at this age (see Table S2). The final sample of 304 EL‐siblings (46.7% girls) was distributed as follows: Sweden (*n* = 140), UK (*n* = 77), Belgium (*n* = 52), the Netherlands (*n* = 35). The number of children with a CBE autism diagnosis at 36 m per site was: Sweden (*n* = 36), UK (*n* = 12), Belgium (*n* = 13), the Netherlands (*n* = 10). For 95 participants, data were available at all three time points, 97 at 14 m and 24 m, 296 at 24 m and 36 m, and 101 at 14 m and 36 m.

### Measures

#### Autism Diagnostic Observation Schedule‐2

The ADOS‐2 (Lord et al., 2012) is a standardised, semi‐structured observational measure designed to assess autism‐related features in communication, social interaction, play and restricted, repetitive or sensory behaviours and interests. In this study, the Toddler Module, Module 1 or Module 2 were administered based on the standard age and language ability criteria specified in the manual (see Table S3). The exception to this was that at 14 m the ADOS Toddler Module was administered if the child could move independently; but 27 children had not yet achieved independent walking at that time. Observations were performed by ADOS‐trained researchers, who met standardised administrations and scoring reliability requirements. ADOS‐2 scores include the Calibrated Severity Score (CSS), Social Affect Score (CSS‐SA) and Restricted and Repetitive Behaviour Score (CSS‐RRB), ranging from 1 to 10. CSS‐values of ≥4 indicate an ADOS‐positive classification for autism spectrum (Gotham, Pickles, & Lord, 2009). CSS‐values for the ADOS‐T were calculated per Esler et al. (2015), while those for Module 1 and 2 per Hus, Gotham, and Lord (2014).

#### Social Responsiveness Scale (SRS)

The SRS (Constantino & Gruber, 2012) is a 65‐item parent‐report questionnaire assessing autistic characteristics over the past 6 months. Items are rated on a 4‐point scale, with higher scores indicating greater social communication difficulties. SRS raw scores were used, with higher scores reflecting more severe social impairments.

#### Mullen Scales of Early Learning

The MSEL (Mullen, 1995) assesses verbal and non‐verbal skills in children aged 1–68 m across four domains: fine motor, visual perception, receptive language and expressive language. *T*‐scores and percentiles were calculated, with the Early Learning Composite (ELC; *M* = 100, *SD* = 15) reflecting overall cognitive ability. Trained researchers, meeting 90% scoring reliability through role‐play and video scoring, conducted the assessments. Experienced clinicians validated all assessments.

#### Clinical Best Estimate Diagnosis of Autism

CBE outcome, based on DSM‐5 criteria (APA, 2013), was determined at 36 m through clinical consensus, the gold standard for diagnosing autism (Bishop & Lord, 2023). Decisions integrated ADOS‐2 results, diagnostic interviews (i.e., ADI‐R) and parent‐reported screeners but were also informed by item‐level or qualitative information, developmental assessments, researcher observations made during the assessments and additional parent‐reported information. All diagnoses were made by a team including at least one experienced senior clinical psychologist and a researcher involved in the assessments, both with expertise in young children with developmental disorders. At least one team member remained consistent across all CBE assessments.

#### Maternal education

Maternal education, coded from 1 (primary) to 4 (university) was used to characterise the sample. It approximates socioeconomic status but reflects only part of this broad construct (Duncan & Magnuson, 2005).

Sample descriptives are shown in Table 1.

**Table 1:** EL‐children characteristics

	CBE autism		CBE non‐autism		Test	*p*‐Value	α*
	*n* = 71 (23.4%)		*n* = 233 (76.6%)				
	Frequency (%)		Frequency (%)				
Sex	26 female (36.6%)		116 female (49.8%)		*χ* ^2^ = 3.79	.052	
	45 male (63.4%)		117 male (50.2%)				
Maternal education					*χ* ^2^ = 3.06	.383	
1: Primary	2 (2.8%)		4 (1.7%)				
2: Secondary	21 (29.6%)		48 (20.6%)				
3: Tertiary‐undergraduate	18 (25.4%)		67 (28.8%)				
4: Tertiary‐postgraduate	21 (29.6%)		83 (35.6%)				
Missing	9 (12.6%)		31 (13.3%)				
	**Mean (SD)**	**Range**	**Mean (SD)**	**Range**			
Age (months)							
14‐month visit	14.34 (0.53)	13.34–15.93	14.40 (0.67)	12.98–16.95	*U* = 7,244	.694	
24‐month visit	24.87 (1.12)	23.20–29.47	24.93 (1.21)	23.16–30.63	*U* = 7,946	.870	
36‐month visit	38.20 (2.59)	35.57–47.28	37.73 (2.25)	35.53–48.79	*U* = 6,982.50	.233	
ELC (MSEL)							
14‐month visit	86.15 (13.93)		91.57 (14.79)		*U* = 5,708	.**007**	.05
24‐month visit	85.64 (19.20)		99.42 (16.54)		*U* = 3,928.50	**<.001**	.017
36‐month visit	91.34 (23.22)		105.37 (17.58)		*U* = 4,376	**<.001**	.025

Maternal education = highest achieved diploma of participant's mother, α*: adjusted alpha level determining significance after Holm‐Bonferroni correction was applied; Significant differences are marked in bold. CBE, Clinical Best Estimate; ELC, Early Learning Composite; MSEL, Mullen Scales of Early Learning; SD, standard deviation.

### Data analysis

Descriptive analyses calculated the number of EL‐siblings with ADOS‐positive scores at the different time points. Changes in ADOS‐positive proportions (14–36 m) were assessed using Cochran's *Q*‐test, with post‐hoc pairwise comparisons using Dunn's procedure and Bonferroni correction (Abdi, 2007; Dunn, 1964). Stability coefficients measured the proportion of children maintaining an ADOS‐2 autism (spectrum) classification over time. Mann–Whitney *U* tests compared ADOS‐2 CSS (total/SA/RRB) between children with and without a CBE autism diagnosis, with Holm‐Bonferroni correction for multiple testing (Holm, 1979). Chi‐squared test analysed sex and site differences in ADOS‐2 classification and stability. Given variation in CBE diagnosis across sites, site differences in ADOS‐2 classification were analysed separately for the CBE autism and CBE non‐autism groups. Mann–Whitney *U* tests were used to compare MSEL scores across ADOS‐2 classifications at each timepoint. Group differences in MSEL‐ and SRS‐scores between children with stable positive, stable negative and unstable ADOS‐2 classifications were examined using a Kruskal–Wallis *H* test, followed by post‐hoc pairwise comparisons with Bonferroni correction. Sensitivity, specificity, positive predictive value (PPV) and negative predictive value (NPV) of ADOS‐2 classifications at 14, 24 and 36 m compared to CBE autism diagnosis at 36 m were calculated. A sensitivity analysis excluding non‐walking children at 14 m is reported in Table S4. Analyses were conducted using SPSS version 28 (IBM Corp., 2021).

## Results

At 36 m 71 from 304 EL‐siblings (23.4%) met DSM‐5 CBE diagnostic criteria for autism. This comprised 45 from 161 boys (28.0%) and 26 from 142 (18.1%) girls, a marginally significant sex difference (*χ* ^2^(1) = 3.79, *p* = .052).

### ADOS‐2 classification at 14, 24 and 36 months

A total of 40 children (38.8%; 95% CI [29%, 48%]) received an ADOS‐positive classification at 14 m. No significant sex (*χ* ^2^(1) = 1.36, *p* = .244) or site (CBE no autism: *χ* ^2^(2) = 1.97, *p* = .37); CBE autism: (*χ* ^2^(2) = 3.88, *p* = .144) differences were found, see Table 2 and S4. Children with ADOS‐2 positive classifications had significantly lower MSEL‐ELC scores (*U* = 637.5, *p* < .001), see Table S5.

**Table 2:** Site differences in ADOS‐2 classifications

Site	CBE autism			*p*‐Value	CBE non‐autism			*p*‐Value
	ADOS‐positive	ADOS‐negative			ADOS‐positive	ADOS‐negative		
	*n*	*n*			*n*	*n*		
14 months			*χ* ^2^ = 3.88	.144			*χ* ^2^ = 1.97	.37
Belgium	6	7			10	27		
Netherlands	0	2			1	7		
Sweden	10	3			11	19		
24 months			*χ* ^2^ = 5.83	.120			*χ* ^2^ = 12.13	.**007**
Belgium	11	1			8	30		
Netherlands	6	4			3	22		
Sweden	26	9			43	58		
UK	6	6			19	46		
36 months			*χ* ^2^ = 25.64	**<.001**			*χ* ^2^ = 18.66	**<.001**
Belgium	13	0			15	24		
Netherlands	8	2			3	22		
Sweden	35	1			43	59		
UK	5	7			10	55		

Significant differences are marked in bold. CBE, Clinical Best Estimate.

A total of 122 children (40.9%; 95% CI [35%, 46%]) received an ADOS‐positive classification at 24 m. No significant sex differences were found (*χ* ^2^(1) = 1.69, *p* = .194), see Table S5. In the CBE no autism group site differences were significant (*χ* ^2^(3) = 12.13, *p* = .007), but not in the autism group (*χ* ^2^(3) = 5.83, *p* = .120), see Table 2. Children with ADOS‐2 positive classifications had significantly lower MSEL‐ELC scores (*U* = 6,619, *p* < .001), see Table S5.

A total of 132 children (43.7%; 95% CI [38%, 49%]) received an ADOS‐positive classification at 36 m. No significant sex differences were found (*χ* ^2^(1) = 3.15, *p* = .082). In both the CBE no autism and autism groups, significant site differences were found (respectively: *χ* ^2^(3) = 18.66, *p* < .001; *χ* ^2^(3) = 25.64, *p* < .001). See Table 2 and S4. Children with ADOS‐2 positive classifications had significantly lower MSEL‐ELC scores (*U* = 8,146, *p* < .001); see Table S5.

Cochran's *Q* test showed a significant difference in ADOS‐positive proportions across different time points (*χ* ^2^(2) = 9.529, *p* = .009). Post‐hoc Dunn's tests indicated no significant increase from 14 to 24 m (*p* = .368) and from 24 to 36 m (*p* = .368); however, there was a significant increase from 14 to 36 m (*p* = .006). The relationship between ADOS‐2 scores and CBE diagnosis of autism is shown in Table S6.

### Stability of ADOS‐2 classification from 14 to 36 months

#### Stability and change from 14 to 24 months

**Figure 1:**
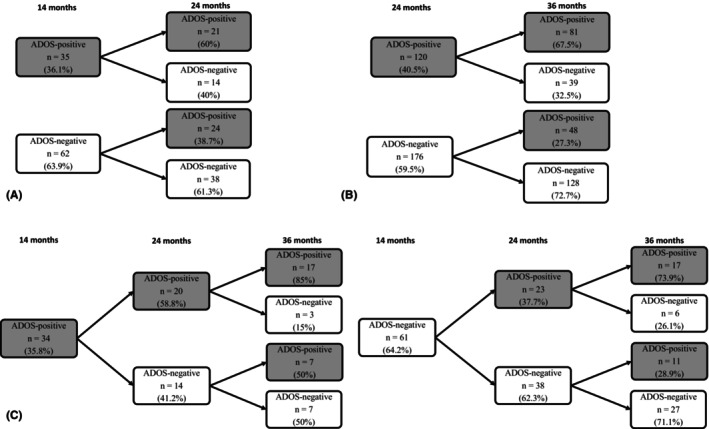
Stability of ADOS‐2 classifications across visits. (A) stability from 14 to 24 months, (B) stability from 24 to 36 months, (C) stability from 14 to 36 months

Of the 35 siblings with an ADOS‐positive result at 14 m, 21 retained this classification at 24 m, which resulted in a stability coefficient of 60%. 38.7% of the siblings with no ADOS‐positive classification at 14 m met the cut‐off at the 24 m assessment (see Figure 1a).

#### Stability and change from 24 to 36 months

Of the 120 siblings with an ADOS‐positive result at 24 m, 81 retained this classification at 36 m, which resulted in a stability coefficient of 67.5%. 27.3% of the children with no ADOS‐positive classification at 24 m met the cut‐off at the 36 m assessment (see Figure 1b).

#### Stability and change from 14 to 36 months

As shown in Figure 1c, the overall stability of an ADOS‐positive classification from 14 to 36 m was 70.6%, with 24 from 34 siblings retaining their classification over this period. Of these, 17 had stable ADOS‐positive classifications across 14, 24 and 36 m, while 7 had an unstable pattern, being ADOS‐negative at 24 m. Of those siblings who were ADOS‐negative at 14 m, 28 (45.9%) received an ADOS‐positive classification at 36 m.

### Stability patterns of children with a CBE outcome of autism

Table 3 gives an overview of the ADOS‐classification patterns in relation to CBE outcome. At 36 m, out of the 95 EL‐siblings with ADOS‐2 data at all three timepoints 26 (27.4%) were given a CBE clinical diagnosis of autism according to DSM‐5 criteria. More than half (16 out of 26; 61.5%) of these children already had an ADOS‐positive result at 14 m. Furthermore, out of these 16 children, 13 had ADOS‐positive results from 14 m onwards, although three of them had instable ADOS‐classifications, with an ADOS‐positive result at 14 m, but not at 24 m (see Table 3). At 24 m, almost all children with a CBE diagnosis of autism had an ADOS‐positive result (23 out of 26; 88.5%). All children with a CBE of autism were ADOS‐positive at 36 m. However, 60.9% (42 out of 69) of the children with no CBE of autism had an ADOS‐positive result at one or more time points by the age of 3 . An overview of the ADOS‐classification patterns at 24 and 36 m in relation to CBE per site can be found in Tables S7–S11. No significant sex differences were found between children with stable‐positive, stable‐negative and unstable ADOS‐2 classifications over time (*χ* ^2^(2) = 3.083, *p* = .214). Mullen ELC scores were significantly higher in children without CBE of autism at all timepoints (*U* = 5,708, *p* = .007; *U* = 5928.5, *p* < .001; *U* = 4,376, *p* < .001 respectively, see also Table 1). There were significant differences in MSEL scores between children with stable‐positive, stable‐negative and unstable ADOS‐2 classification patterns at all time points (14 m: *H*(2) = 11.93, *p =* .003; 24 m: *H*(2) = 13.54, *p =* .001; 36 m: *H*(2) = 16.93, *p* < .001). Post‐hoc comparisons with Bonferroni correction showed that children with stable‐positive classifications had significantly lower scores than those with stable‐negative classifications (14 m: *p =* .004, 24 m: *p = .001*, 36 m: *p <* .001) and unstable ADOS‐2 classifications (14 m: *p =* .005, 24 m: *p = *.004, 36 m: *p =* .006) at all timepoints. Children with stable‐negative classifications did not differ from children with unstable classifications at any timepoint (14 m: *p =* 1, 24 m: *p =* 1, 36 m: *p =* .296), see Table S12. There were no significant differences in SRS scores between children with stable‐positive, stable‐negative and unstable ADOS‐2 classification patterns (*H*(2) = 4.03, *p* = .133), see Table S12. To further explore the variability between ADOS‐2 classification and CBE diagnosis, we have plotted the ADOS CSS‐trajectories of children with fluctuating ADOS‐2 classifications per child, split by CBE outcome (autism vs. no autism; see Figure S1). This includes children with ADOS‐positive classifications at 36 m but who showed inconsistent classifications at 14 and 24 m.

**Table 3:** ADOS‐classification patterns in relation to Clinical Best Estimate diagnosis of autism

ADOS‐classification			Clinical Best Estimate		Total
14 months	24 months	36 months	CBE autism	CBE no autism	
			*n* = 26	*n* = 69	*n* = 95
			13 (50%)	4 (5.8%)	17
			0	3 (4.3%)	3
			0	7 (10.2%)	7
			3 (11.5%)	4 (5.8%)	7
			0	6 (8.7%)	6
			10 (38.5%)	7 (10.2%)	17
			0	11 (15.9%)	11
			0	27 (39.1%)	27

Classification, green ADOS‐positive, red ADOS‐negative. CBE, Clinical Best Estimate.

The psychometric properties of the ADOS‐2 classifications at 14, 24 and 36 m are presented in Table 4.^1^ Sensitivity increased significantly with age, from 64% at 14 m to 86% at 36 m (*χ* ^2^(2) = 12.154, *p* = .002), with post‐hoc McNemar tests showing a significant increase between 14 and 36 m, and additionally between 24 and 36 m using Holm correction (Holm, 1979). Specificity and PPV remained relatively stable across time points, while NPV showed a descriptive increase (from 84% at 14 m to 94% at 36 m), though not statistically significant.

**Table 4:** Sensitivity, specificity PPV and NPV of the ADOS‐2 as compared to CBE diagnosis

Instrument	No. of positive cases		No. of negative cases		Sensitivity	Specificity	PPV	NPV
	True positive	False positive	True negative	False negative	(95% CI)	(95% CI)	(95% CI)	(95% CI)
ADOS 14 m^a^	18	22	53	10	64.3% (45.9–80.2)	70.7% (59.8–80.2)	45% (30.3–60.4)	84.1% (73.8–91.7)
ADOS 24 m^a^	49	73	156	20	71% (59.7–80.8)	68.1% (61.9–73.9)	40.2% (31.7–49)	88.6% (83.4–92.8)
ADOS 36 m^a^	61	71	160	10	85.9% (76.6–92.7)	69.3% (63.1–75)	46.2% (37.8–54.7)	94.1% (89.9–97)

CBE, Clinical Best Estimate; NPV, negative predictive value; PPV, positive predictive value; No. total number. 14 m timepoint at 14 months, 24 m timepoint at 24 months, 36 m timepoint at 36 months.

^a^ An ADOS‐2 classification of autism requires meeting or exceeding the autism spectrum cut‐point. The cut‐point for the Toddler Module is a total score ≥10 for children between 10 and 20 months old or between 21 and 30 months with little‐to‐no words or ≥8 for children between 21 and 30 months with some words. The cut‐point for Module 1 is a total score of ≥11 (no‐to‐few words) or ≥8 (some words). The cut‐point for Module 2 is a total score of ≥7.

## Discussion

In this prospective longitudinal study, we examined the stability of ADOS‐2 autism spectrum classifications across three early childhood timepoints in EL‐siblings. EL‐siblings are an important population to study, as the presence of an autism diagnosis in an older sibling provides a natural context to monitor younger siblings from an early age using standardised tools such as the ADOS‐2. To our knowledge, this is the first investigation to examine whether the ADOS‐2, a well‐established observational diagnostic instrument, provides a stable and reliable identification of autism in EL‐siblings prior to the age of three.

The recurrence rate of 23.4% and the (marginally) higher rate in males than females are consistent with prior reports in EL‐sibling samples (Ozonoff et al., 2024). Our results show that for some EL‐children, ADOS‐2 classifications are stable from early on. The stability of ADOS‐positive classifications at 14 and 24 m relative to the classification at 36 m was moderate (70.6% and 67.5%, respectively). The majority of the ADOS‐2 classifications made before 36 m, were confirmed at 36 m (see Figure 1). For a subgroup of children however, a classification at one point in time was inconsistent with their classification at subsequent timepoints. Approximately 30% of the sample with unstable patterns received ADOS‐positive classifications at 14 m and/or 24 m, and ADOS‐negative classifications at age 3. Several factors may account for this discrepancy; one possibility is that EL‐siblings exhibit transient behaviours in early childhood, which may not persist over time (Gamliel, Yirmiya, & Sigman, 2007). Another possibility is misclassification due to measurement error, especially near the diagnostic threshold, as well as overlap with other behaviours, such as shyness and anxiety or characteristics of developmental delay or ADHD that can impact ADOS score (Greene, Vasile, Bradbury, Olsen, & Duvall, 2021; Hamrick, Ros‐Demarize, Kanne, & Carpenter, 2024; Hayashi et al., 2022). Module selection may also affect stability. Administering higher modules with more demanding tasks, when children only just qualify for them, may result in higher ADOS‐2 scores, whereas administering lower modules may result in lower scores (Klein‐Tasman, Risi, & Lord, 2007). Differences in classifications at 24 m may partly reflect differences between the Toddler Module and Module 2, since the five children without a CBE autism diagnosis who met the ADOS‐2 cutoff on Module 2, only did so at this timepoint. These variations likely capture developmental shifts rather than measurement error; see Table S3.

The stability of an ADOS‐negative classification was lower, approximately 55% at 14 m and about 73% at 24 m relative to the ADOS‐2 assessment at 36 m. As PPV and specificity remained relatively stable across timepoints (see Table 4), this pattern likely reflects increasing diagnostic clarity with age rather than a rise in false positives. Sensitivity and specificity were generally moderate (Glascoe, 2005). Sensitivity increased with age, indicating that the ADOS‐2 better identifies children with autism as they grow older, although the good sensitivity at 36 m may be partly reflective of the incorporation of the ADOS‐2 in the CBE. Specificity remained relatively stable across time. These estimates are somewhat lower than those reported in clinical samples (e.g., Lebersfeld, Swanson, Clesi, & O'Kelley, 2021), likely due to differences in referral context and autism prevalence. NPV was consistently high, while PPV remained low across ages, suggesting limited predictive value of a positive ADOS‐2 classification in EL‐siblings up to 3 years. As few studies have reported PPV in this population, direct comparisons are limited. This underscores the complexity of early identification and the importance of continued follow‐up, even in children who do not yet display clear autism characteristics, as some may not meet ADOS‐2 classification thresholds, or receive a CBE, until or after age 3 (Bazelmans et al., 2024; Brian et al., 2016; Ozonoff et al., 2018).

Direct comparison is challenging given the absence of prior research examining the stability of ADOS‐2 classification at such a young age. The most comparable studies are those assessing diagnostic stability over time in EL‐siblings (Ozonoff et al., 2015; Zwaigenbaum et al., 2016), albeit with a significant distinction; these studies established a diagnosis at each age with a more comprehensive evaluation, whereas an ADOS‐positive classification was not a prerequisite for diagnosis. As might be expected, these studies that rely on multiple instruments and sources of information and expert consensus diagnostic opinion show higher stability (Ozonoff et al. (2015): 82%–93%; Zwaigenbaum et al. (2015): 82.6%) than we found based on a single observational instrument the ADOS‐2. ADOS‐2 assessments capture a momentary snapshot and are highly dependent on circumstances and the child's response during a time‐limited assessment often in an unfamiliar context. They provide only limited information on restricted and repetitive and sensory behaviours, necessitating complementary information to determine a CBE diagnosis (Kim & Lord, 2010). Therefore, the higher stability observed in studies that assess diagnostic stability, compared to our results is not surprising.

Moreover, an autism diagnosis requires more than scoring on a standardised instrument. Best practices emphasise the need for comprehensive assessment combining information from multiple sources including parents and direct observations, with instruments like the ADOS‐2 serving as informative, but not definitive components (Bishop & Lord, 2023; Fombonne, 2023). While it may be possible to identify developmental concerns early in some EL‐siblings, ongoing monitoring and surveillance remain essential, particularly when characteristics are ambiguous (Ozonoff et al., 2011; Zwaigenbaum et al., 2021). This process may include repeated assessments, such as the ADOS‐2, alongside parental interviews and other clinical information (Zwaigenbaum et al., 2015).

Examining patterns of EL‐siblings with a CBE outcome of autism at 36 m also revealed that the stability of ADOS‐positive classifications increased with age suggesting that for some children the ADOS‐2 is already informative early on. In the subgroup with ADOS‐2 data at all timepoints (*n* = 95), all EL‐ siblings with an autism CBE were ADOS‐positive at 36 m. However, the ADOS‐2 also classified some non‐autistic children as positive, underscoring the need for clinical judgement (see Table 3). The agreement between ADOS‐2 classifications and CBE diagnosis varied across sites. This variability may reflect differences in sample characteristics, ADOS‐2 scoring or diagnostic differences, factors we could not formally assess. Given language, practical and resource constraints of this multi‐national study we did not attempt any cross‐site diagnostic consensus process. Although variability in ADOS‐2 classification patterns was observed in both groups (autism vs. no autism) in our sample, no clear pattern emerged (see Figure S1). No sex differences were found in ADOS‐2 classification patterns. Despite efforts to minimise site differences in ADOS‐2 scoring through training and reliability meetings, some time point‐specific variability was observed though it was not consistent over time. These findings underscore the need to interpret early ADOS‐2 classifications within a broader clinical context. Additionally, it is important to consider that the ADOS‐2 at 36 m was taken into account when determining the CBE diagnosis, which introduces bias into the assessment of agreement. Our results suggest that, for some children, ADOS‐2 classifications are already stable and accurate before age 3 and may be informative as early as 14 m of age. Although we found no evidence for more pronounced autism characteristics in this group, children with stable positive ADOS‐2 classification consistently showed lower developmental levels across all timepoints.

However, caution is needed, as approximately 60% of those without a CBE of autism scored above the ADOS autism spectrum cut‐off at least once up to age 3. It is challenging to determine why these children score above the ADOS cut‐off yet do not receive a CBE diagnosis of autism. One possible explanation is the relationship between ADOS‐2 score and developmental delay, which could lead to children with delayed development being incorrectly identified by the ADOS‐2. In our study children without a CBE diagnosis but with a positive ADOS‐2 classification exhibited statistically significantly lower MSEL scores at 14 and 24 m (see Table S5). However, by 36 m, this difference was no longer significant. Another potential explanation is that certain children receive positive ADOS‐2 results driven by social communication difficulties, yet an autism diagnosis requires the presence of both social communicative difficulties and restricted, repetitive behaviours and interests and sensory differences. The ADOS‐2 provides only limited information on repetitive and sensory behaviours, necessitating complementary information to determine a CBE diagnosis (Kim & Lord, 2010). Furthermore, observed behaviours may not cause clinically significant challenges in everyday functioning, which is a requirement for diagnosis (APA, 2022). Lastly, these children may not meet the criteria for an autism diagnosis at age 3 but may do so later in their development (Bazelmans et al., 2024; Brian et al., 2016).

## Limitations and directions for future research

While this study provides valuable insights into the stability of the ADOS‐2 classifications in EL‐siblings, several limitations and future directions should be noted. First, the sample of highly educated mothers limits generalisability. Second, a substantial number of children (*n* = 128) were excluded due to missing or insufficient ADOS‐2 data across time points, potentially impacting representativeness. Third, there was a considerable amount of missing data at 14 m, and 27 siblings were not yet walking independently at their ADOS‐2 assessment, which deviates from standard administration guidelines. However, of these siblings, only two without CBE of autism exceeded the cutoff solely at this point, suggesting minimal impact of motor development on early classifications in our sample. Fourth, potential interventions were not accounted for. Furthermore, site differences may exist between CBE autism diagnostic decision‐making in this multi‐site study, despite being conducted by experienced teams led by expert clinicians using similar methods. Importantly, participants in this study were assessed in the context of a longitudinal research project, rather than referred for diagnostic evaluation. As such, expectations surrounding diagnosis may differ from clinical practice and may also be influenced by parent's knowledge of their older autistic child, potentially influencing the variability between CBE diagnostic thresholds and ADOS scores across sites. Moreover, the relatively small number of children with a CBE autism diagnosis, particularly when broken down by site, limits generalisability. Finally, the absence of agreed criteria for broader autism phenotype (BAP) precluded further subdivision of the non‐autism CBE group into BAP and typically developing subgroups. Future research could (1) examine ADOS trajectories within these subgroups and (2) explore whether CSS scores over time reveal distinct developmental profiles.

## Conclusion

This study demonstrates that, while the ADOS‐2 can offer valuable information to the early monitoring of EL siblings, its stability before age 3 shows notable variability between individuals. A significant proportion of children without a CBE of autism received positive ADOS‐2 classifications at one or more timepoints, highlighting the potential for false positives. These findings underscore the importance of interpreting early ADOS‐2 outcomes with caution and always within the context of broader clinical assessment. Diagnostic decisions should not be based solely on ADOS‐2 results, particularly in very young children. Continued developmental surveillance, incorporating multiple sources of information over time, remains essential for accurate identification. Further research with longer follow‐up periods is needed to clarify which early behavioural features are associated with false positive or false negative ADOS‐2 outcomes and to advance our understanding of the developmental trajectories of autism.

## Ethical considerations

Ethical approval was obtained from local ethics committees in the participating countries. Specifically, approval was granted by the National Health Service REC London Central (reference 13/LO/0751, 29 July 2013) for the UK site; the Regional Ethics Board in Stockholm (reference 2010/2085‐31/3, 23 March 2011) for the Swedish site; the CMO Region Arnhem‐Nijmegen (reference NL42726.091.13, 3 September 2013) for the Dutch sites in Nijmegen and Utrecht; and the ethical board of the Faculty of Psychology and Educational Sciences of Ghent University Ghent (reference: 2011/41, 2014/33, 2015/10 and 2015/81; 24 August 2011, 4 July 2014, 27 February 2015, 29 October 2015). The study was conducted in accordance with the Declaration of Helsinki and the ethical principles of the American Psychological Association. Written informed consent was obtained from parents prior to participation.

**Key points**

**What's known?**

- The ADOS‐2 is widely used to assess autism characteristics in young children, including siblings at elevated likelihood for autism. However, the stability of ADOS‐2 classifications across early childhood remains unclear.

**What's new?**

- ADOS‐2 classification stability increases with age but remains moderate at 14 and 24 months compared to 36 months. A notable proportion of children without clinical best estimate diagnosis of autism showed positive ADOS‐2 results at one or more early time points.

**What's relevant?**

- These finding inform clinical practice by reinforcing the importance of integrating ADOS‐2 results with broader clinical evaluations and longitudinal follow‐up of elevated likelihood siblings to reduce the over‐reliance on single‐time‐point ADOS‐2 results.

## Supporting information


**Table S1.** Available data for the ADOS‐2 per time point.
**Table S2.** MSEL and sex of included and excluded children.
**Table S3.** ADOS‐2 modules and classifications per age and per CBE outcome.
**Table S4.** Sensitivity, specificity PPV and NPV of the ADOS‐2 as compared to CBE diagnosis excluding non‐walking children.
**Table S5.** Sex and developmental differences in ADOS‐2 classifications.
**Table S6.** Difference in ADOS‐2 scores between EL‐children with and without a CBE diagnosis of autism.
**Table S7.** ADOS‐classification patterns at 24 and 36 months in relation to Clinical Best Estimate diagnosis of autism.
**Table S8.** ADOS‐classification patterns at 24 and 36 months in relation to Clinical Best Estimate diagnosis of autism (Belgium).
**Table S9.** ADOS‐classification patterns at 24 and 36 months in relation to Clinical Best Estimate diagnosis of autism (The Netherlands).
**Table S10.** ADOS‐classification patterns at 24 and 36 months in relation to Clinical Best Estimate diagnosis of autism (Sweden).
**Table S11.** ADOS‐classification patterns at 24 and 36 months in relation to Clinical Best Estimate diagnosis of autism (UK).
**Table S12.** Sex, Developmental and SRS Differences in Stable‐Positive, Stable‐Negative and unstable ADOS‐2 Classification patterns.
**Figure S1.** ADOS CSS trajectories of children with fluctuating ADOS‐2 classifications and ADOS‐positive classification at 36 months.

## Data Availability

The data sets generated and analysed during this study are available from the corresponding author upon reasonable request.

## Endnote

1 Sensitivity, specificity, PPV and NPV for the Toddler Module did not substantially differ when non‐walking children were excluded (see Table S4).
