# Supplementary material for: The stability of the Autism Diagnostic Observation Schedule‐2 in children aged 14–36 months with elevated likelihood for autism
Source: J Child Psychol Psychiatry. 2025 Nov 16;67(6):869–80. doi: 10.1111/jcpp.70078 (PMC13170631; doi:10.1111/jcpp.70078)
Supplement: Supplementary file 1 — Table S1. Available data for the ADOS‐2 per time point. Table S2. MSEL and sex of included and excluded children. Table S3. ADOS‐2 modules and classifications per age and per CBE outcome. Table S4. Sensitivity, specificity PPV and NPV of the ADOS‐2 as compared to CBE diagnosis excluding non‐walking children. Table S5. Sex and developmental differences in ADOS‐2 classifications. Table S6. Difference in ADOS‐2 scores between EL‐children with and without a CBE diagnosis of autism. Table S7. ADOS‐classification patterns at 24 and 36 months in relation to Clinical Best Estimate diagnosis of autism. Table S8. ADOS‐classification patterns at 24 and 36 months in relation to Clinical Best Estimate diagnosis of autism (Belgium). Table S9. ADOS‐classification patterns at 24 and 36 months in relation to Clinical Best Estimate diagnosis of autism (The Netherlands). Table S10. ADOS‐classification patterns at 24 and 36 months in relation to Clinical Best Estimate diagnosis of autism (Sweden). Table S11. ADOS‐classification patterns at 24 and 36 months in relation to Clinical Best Estimate diagnosis of autism (UK). Table S12. Sex, Developmental and SRS Differences in Stable‐Positive, Stable‐Negative and unstable ADOS‐2 Classification patterns. Figure S1. ADOS CSS trajectories of children with fluctuating ADOS‐2 classifications and ADOS‐positive classification at 36 months. [file JCPP-67-869-s001.docx]

**The stability of the Autism Diagnostic Observation Schedule-2 in children aged 14 to 36 months with elevated likelihood for autism**

**Supporting information**

**Table S1**

| Available Data for the ADOS-2 per Time Point | | | | | |  |
| --- | --- | --- | --- | --- | --- | --- |
|  | **CBE autism** | | **CBE non-autism** | |  |  |
| **Instrument** | **n** | **Missing (%)** | **n** | **Missing (%)** | | |
| **ADOS 14m**  Belgium  Netherlands  Sweden  UK | 28  13  2  13  0 | 43 (60.6)  0  8 (80)  23 (63.9)  12 (100) | 75  37  8  30  0 | 158 (67.8)  2 (5.1)  17 (68)  74 (71.2)  65 (100) | | |
| **ADOS 24m**  Belgium  Netherlands  Sweden  UK | 69  12  10  35  12 | 2 (2.8)  1 (7.7)  0  1 (2.7)  0 | 229  38  25  101  65 | 4 (1.7)  1 (2.6)  0  3 (2.9)  0 | | |
| **ADOS 36m**  Belgium  Netherlands  Sweden  UK | 71  13  10  36  12 | 0 (0)  0  0  0  0 | 231  39  25  102  65 | 2 (0.9)  0  0  2 (1.9)  0 | | |
| **Participants with data at 14 and 24m**  Belgium  Netherlands  Sweden  UK | 26  12  2  12  0 | 45 (63.4)  1 (7.7)  8 (80)  24 (66.7)  12 (100) | 71  36  8  27  0 | 162 (69.5)  3 (7.7)  17 (68)  77 (74)  65 (100) | | |
| **Participants with data at 24 and 36m**  Belgium  Netherlands  Sweden  UK  **Participants with data at 14 and 36m**  Belgium  Netherlands  Sweden  UK | 69  12  10  35  12  28  13  2  13  0 | 2 (0.03)  1 (7.7)  0  1 (2.8)  0  43 (60.6)  0  8 (80)  23 (63.9)  12 (100) | 227  38  25  99  65  73  37  8  28  0 | 6 (0.03)  1 (2.6)  0  5 (4.8)  0  160 (68.7)  2 (5.1)  17 (68)  76 (73.1)  65 (100) | | |
| **Participants with data at all timepoints**  Belgium  Netherlands  Sweden  UK | 26  12  2  12  0 | 45 (63.4)  1 (7.7)  8 (80)  24 (66.7)  12 (100) | 69  36  8  25  0 | 164 (70.4)  3 (7.7)  17 (68)  79 (75.9)  65 (100) | | |

*Note:* CBE Clinical Best Estimate

| **Table S2**  MSEL and sex of included and excluded children | | | | |  |  |
| --- | --- | --- | --- | --- | --- | --- |
|  | **included** | | **excluded** | |  | **p-value** |
|  | N | Mean (SD) | N | Mean (SD) |  |  |
| **Sex (female:male)** | 304 (46.7:53.3) |  | 128 (44.5:55.5) |  | *X²* = .172 | .678 |
| **ELC (MSEL)**  14 months visit  24 months visit  36 months visit | 287  283  285 | 90.33 (14.75)  96.30 (18.09)  102.22 (19.84) | 80  47  24 | 90.51 (14.54)  98.43 (14.78)  87.54 (21.69) | *U*=11417.5  *U*=6139  *U*=2129 | .941  .398  **.002** |

MSEL Mullen Scales of Early Learning, Significant differences are marked in bold.

| **Table S3**  ADOS-2 Modules and Classifications per Age and per CBE outcome | | | | | | |
| --- | --- | --- | --- | --- | --- | --- |
| **Age** | **Module** (ADOS-2 classification) | | | | | |
|  | **Total frequency (% of different classifications)** | | | | | |
|  | **CBE autism** | | | **CBE non-autism** | | |
|  | Toddler module  (moderate to severe: mild to moderate) | Module 1  (autism:  autism spectrum) | Module 2  (autism:  autism spectrum) | Toddler module  (moderate to severe: mild to moderate) | Module 1  (autism:  autism spectrum) | Module 2  (autism:  autism spectrum) |
| 14m | 28  (39.3%:25%) | / | / | 75  (13.3%:16%) |  |  |
| 24m | 65 (38.5%:30.8%) | / | 4  (50%:50%) | 209  (10%:20.1%) |  | 20  (10%:40%) |
| 36m | / | 24  (66.7%:25%) | 47  (74.5%:8.5%) |  | 33  (15.1%:27.3%) | 198  (10.6%:18.2%) |

*Note:* CBE Clinical Best Estimate

| **Table S4**  Sensitivity, specificity PPV and NPV of the ADOS-2 as compared to CBE diagnosis excluding non-walking children | | | | | | | | |
| --- | --- | --- | --- | --- | --- | --- | --- | --- |
| Instrument | No. of positive cases | | No. of negative cases | | Sensitivity | Specificity | PPV | NPV |
|  | True positive | False positive | True negative | False negative | (95% CI) | (95% CI) | (95% CI) | (95% CI) |
| ADOS 14m ^a^ | 12 | 17 | 41 | 6 | 66.7%  (43.7-85.2) | 70.7%  (58.3-81.3) | 41.4%  (24.8-59.9) | 87.2%  (75.8-94.7) |
| ADOS 24m ^a^ | 40 | 69 | 143 | 20 | 66.7%  (54.2-77.7) | 67.5%  (61.0-73.5) | 36.7%  (28.0-46.0) | 87.7%  (82.1-92.2) |
| ADOS 36m ^a^ | 51 | 64 | 150 | 10 | 83.6%  (73.0-91.4) | 70.1%  (63.7-76.0) | 44.3%  (35.5-53.5) | 93.8%  (89.3-96.8) |

**Table S5**

Sex and Developmental Differences in ADOS-2 Classifications

|  | **ADOS-positive** |  | **ADOS-negative** |  |  | **p-value** |
| --- | --- | --- | --- | --- | --- | --- |
|  | **n (%)** |  | **n (%)** |  |  |  |
| **Sex (female:male)**  14 month visit  24 months visit  36 months visit | 15:25  (37.5:62.5)  51:71  (41.8:58.2)  54:78 (40.9:59.1) |  | 31:32  (49.2:50.8)  87:89  (49.4:50.6)  87:83  (51.2:48.8) |  | *X²* = 1.36  *X²* = 1.69  *X²* = 3.15 | .244  .194  .082 |
|  | **Mean (SD)** | **range** | **Mean (SD)** | **range** |  |  |
| **ELC (MSEL)**  14 months visit  24 months visit  36 months visit | 87.58 (12.29)  91.12 (18.82)  98.34 (22.22) | 59-109  50-143  49-147 | 99.78 (11.49)  100.13 (16.55)  105.32 (17.25) | 72-123  53-139  61-142 | *U* = 637.5  *U* = 6619  *U* = 8146 | **<.001**  **<.001**  **.011** |

MSEL Mullen Scales of Early Learning, Significant differences are marked in bold.

| **Table S6**  Difference in ADOS-2 scores between EL-children with and without a CBE diagnosis of autism | | | | | | | | | | |  |
| --- | --- | --- | --- | --- | --- | --- | --- | --- | --- | --- | --- |
| **Instrument** | **CBE autism**  **(n = 85)** | | **CBE non-autism**  **(n = 246)** | **p value** | | | | **α*** | | ***PS*** |  |
|  | Mean |  | Mean | |  |  | |  | | |  |
| **ADOS CSS 14m**  Total  SA  RRB | 4.71  5.29  4.82 |  | 3.17  3.36  3.85 | | **<.001**  **<.001**  **.033** | | 0.006  0.006  0.05 | | 0.97  0.97  0.96 | | |
| **ADOS CSS 24m**  Total  SA  RRB | 4.91  4.74  5.62 |  | 2.90  3.16  4.12 | | **<.001**  **<.001**  **<.001** | | 0.007  0.008  0.01 | | 0.77  0.73  0.71 | | |
| **ADOS CSS 36m**  Total  SA  RRB | 5.90  5.8  7.2 |  | 2.73  3.03  4.75 | | **<.001**  **<.001**  **<.001** | | 0.013  0.017  0.025 | | 0.86  0.82  0.82 | | |

*Note:* CBE Clinical Best Estimate, PS Probability of superiority = effect size measure that provides an estimate of the probability that a child with a CBE of autism scores higher than a child without CBE of autism; IQR Interquartile range; 14m = timepoint at 14 months, 24m = timepoint at 24 months, 36m = timepoint at 36 months, ADOS-2 CSS calibrated severity score of the Autism Diagnostic Observation Schedule-Second Edition; SA Social Affect, RRB Restrictive and repetitive behaviours, α* adjusted alpha level determining significance after Holm-Bonferroni correction was applied; Significant differences are marked in bold.

**Table S7**

| ADOS-classification patterns at 24 and 36 months in relation to Clinical Best Estimate diagnosis of autism | | | | | |
| --- | --- | --- | --- | --- | --- |
| **ADOS-classification** | | **Clinical Best Estimate** | |  | |
| 24 months | 36 months | CBE autism  n = 69 | CBE no autism  n = 227 | | Total  n = 296 |
|  |  | 46 (66.7%) | 35 (15.4%) | | 81 |
|  |  | 3 (4.3%) | 36 (15.9%) | | 39 |
|  |  | 13 (18.8%) | 35 (15.4%) | | 48 |
|  |  | 7 (10.2%) | 121 (53.3%) | | 128 |

*Note:* CBE Clinical Best Estimate, classification, green ADOS-positive, red ADOS-negative.

**Table S8**

ADOS-classification patterns at 24 and 36 months in relation to Clinical Best Estimate

diagnosis of autism (Belgium)

| **ADOS-classification** | | **Clinical Best Estimate** | |  | |
| --- | --- | --- | --- | --- | --- |
| 24 months | 36 months | CBE autism  n = 12 | CBE no autism  n = 38 | | Total  n = 50 |
|  |  | 11 (91.7%) | 5 (14.3%) | | 16 |
|  |  | 0 | 3 (7.9%) | | 3 |
|  |  | 1 (8.3%) | 10(26.3%) | | 11 |
|  |  | 0 | 20 (52.6%) | | 20 |

*Note:* CBE Clinical Best Estimate, classification, green ADOS-positive, red ADOS-negative.

| **Table S9**  ADOS-classification patterns at 24 and 36 months in relation to Clinical Best Estimate diagnosis of autism (The Netherlands) | | | | |
| --- | --- | --- | --- | --- |
| **ADOS-classification** | | **Clinical Best Estimate** | |  |
| 24 months | 36 months | CBE autism  n = 10 | CBE no autism  n = 25 | Total  n = 35 |
|  |  | 6 (60%) | 2 (8%) | 8 |
|  |  | 0 | 1 (4%) | 1 |
|  |  | 2 (20%) | 1 (4%) | 3 |
|  |  | 2 (20%) | 21 (84%) | 23 |

*Note:* CBE Clinical Best Estimate, classification, green ADOS-positive, red ADOS-negative.

| **Table S10**  ADOS-classification patterns at 24 and 36 months in relation to Clinical Best Estimate diagnosis of autism (Sweden) | | | | |
| --- | --- | --- | --- | --- |
| **ADOS-classification** | | **Clinical Best Estimate** | | |
| 24 months | 36 months | CBE autism  n = 35 | CBE no autism  n = 99 | Total  n = 134 |
|  |  | 25 (71.4%) | 24 (24.2%) | 49 |
|  |  | 1 (2.9%) | 17 (17.2%) | 18 |
|  |  | 9 (25.7%) | 18 (18.2%) | 27 |
|  |  | 0 | 40 (40.4%) | 40 |

*Note:* CBE Clinical Best Estimate, classification, green ADOS-positive, red ADOS-negative.

| **Table S11**  ADOS-classification patterns at 24 and 36 months in relation to Clinical Best Estimate diagnosis of autism (UK) | | | | | |
| --- | --- | --- | --- | --- | --- |
| **ADOS-classification** | | **Clinical Best Estimate** | | |  |
| 24 months | 36 months | CBE autism  n = 12 | CBE no autism  n = 65 | Total  n = 77 | |
|  |  | 4 (33.3%) | 4 (6.2%) | 8 | |
|  |  | 2 (16.7%) | 15 (23.1%) | 17 | |
|  |  | 1 (8.3%) | 6 (9.2%) | 7 | |
|  |  | 5 (41.7%) | 40 (61.5%) | 45 | |

*Note:* CBE Clinical Best Estimate, classification, green ADOS-positive, red ADOS-negative.

| **Table S12**  Sex, Developmental and SRS Differences in Stable-Positive, Stable-Negative and unstable ADOS-2 Classification patterns | | | | | |
| --- | --- | --- | --- | --- | --- |
|  | **Stable ADOS-positive** | **Stable ADOS-negative** | **Unstable ADOS classifications** |  | **p-value** |
|  | **n (%)** | **n (%)** |  |  |  |
| **Sex**  (female:male) | 8:9  (47:53) | 15:12  (56:44) | 18:33  (35:65) |  | .214 |
|  | **Mean (SD)** | **Mean (SD)** | **Mean (SD)** |  |  |
| **MSEL ELC**  14 months visit  24 months visit  36 months visit | 84.88 (13.23)  80.76 (17.89)  83.18 (22.03) | 99.11 (10.17)  102.74 (15.74)  111.33 (16.92) | 97.43 (12.93)  98.80 (17.81)  104.06 (19.14) | *H* = 11.93  *H* = 13.54  *H* = 16.93 | **.003**  **.001**  **<.001** |
| **SRS** | 56.92 (41.24) | 32.50 (23.66) | 38.21 (23.12) | *H* = 4.03 | **.**133 |

MSEL Mullen Scales of Early Learning, SRS Social Responsiveness Scale, Significant differences are marked in bold.

**Figure S1**

ADOS CSS trajectories of children with fluctuating ADOS-2 classifications and ADOS-positive classification at 36 months

*Note: CSS* Calibrated Severity Score

Solid line: EL-siblings without CBE autism; Dashed line: EL-siblings with CBE autism; Red

Line: CSS 4 or cut point for ADOS-2 classification of autism

*CSS_14m* CSS at 14 months, *CSS*_*24m* CSS at 24 months, *CSS*_*36m* CSS at 36 months
